# Coffee, smoking and aspirin are associated with age at onset in idiopathic Parkinson’s disease

**DOI:** 10.1007/s00415-022-11041-x

**Published:** 2022-03-02

**Authors:** Carolin Gabbert, Inke R. König, Theresa Lüth, Beke Kolms, Meike Kasten, Eva-Juliane Vollstedt, Alexander Balck, Anne Grünewald, Christine Klein, Joanne Trinh

**Affiliations:** 1 Institute of Neurogenetics, University of Lübeck, Ratzeburger Allee 160, 23538 Lübeck, Germany; 2 Institute of Medical Biometry and Statistics, University of Lübeck, Lübeck, Germany; 3 Department of Psychiatry and Psychotherapy, University of Lübeck, Lübeck, Germany; 4 Luxembourg Centre for Systems Biomedicine, University of Luxembourg, Esch-sur-Alzette, Luxembourg

**Keywords:** Parkinson’s disease, Age at onset, Modifiers, Lifestyle, Environment

## Abstract

Parkinson’s disease (PD) is a progressive neurodegenerative disorder. Genetic modifiers, environmental factors and gene–environment interactions have been found to modify PD risk and disease progression. The objective of this study was to evaluate the association of smoking, caffeine and anti-inflammatory drugs with age at onset (AAO) in a large PD cohort. A total of 35,963 American patients with idiopathic PD (iPD) from the Fox Insight Study responded to health and lifestyle questionnaires. We compared the median AAO between different groups using the non-parametric Mann–Whitney *U* test. Non-parametric Spearman’s correlation was used for correlation assessments and regression analysis was used to assess interaction between variables. We found that smoking (*p* < 0.0001), coffee drinking (*p* < 0.0001) and aspirin intake (*p* < 0.0001) show an exploratory association with AAO in PD, that was further supported by multivariate regression models. The association of aspirin with PD AAO was replicated in another cohort (EPIPARK) (*n* = 237 patients with PD).

**Supplementary Information:**

The online version contains supplementary material available at 10.1007/s00415-022-11041-x.

## Introduction

Parkinson’s disease (PD) is a progressive neurodegenerative disorder, characterized by dopaminergic neuronal loss in the substantia nigra and the presence of Lewy Bodies [1, 2]. It is the second-most common neurodegenerative disorder and the fastest-growing neurological disease currently affecting over 7 million patients worldwide [3].

A phenomenon in PD is variable age at onset (AAO) that is considered a consequence of genetic and environmental factors. Tobacco use and smoking are already known protective factors for PD risk [4–6]. However, research specifically on AAO is not as extensive. Studies report that disease onset in patients with idiopathic or monogenic PD is later among smokers, dependent on the dosage [7–13]. The largest cross-sectional cohort was comprised of 715 PD patients, of whom 312 were smokers and 404 never smoked [5]. Likewise, caffeine consumption was associated with lower PD risk, with a dosage-dependent level of protection [14]. In terms of AAO, there is evidence that the onset of PD among coffee drinkers is later compared to non-drinkers [11, 15, 16], also indicating a dosage effect [12, 17]. However, earlier studies report opposing effects of an earlier AAO with higher coffee intake [7]. Non-steroidal anti-inflammatory drug (NSAID) intake has been found to be associated with a lower risk for PD [18], supporting work that describe a role for neuro-inflammatory signaling in PD [19]. NSAIDs (ibuprofen and aspirin) have been found to influence the penetrance of *LRRK2* [20]. However, there are currently no studies published that investigate an association between aspirin and AAO in idiopathic PD (iPD).

Herein, we focused on lifestyle factors implicated in PD risk and investigated the association of smoking, the consumption of caffeine and the use of aspirin on AAO in patients with iPD. We hypothesize these factors are associated with AAO in a large cohort of American iPD patients (*n* = 35,963).

## Methods

### Demographics and participant examination

Our study is composed of 35,963 American patients with PD (Table S1) from the Fox Insight Study (Supplementary text and Fig. S1). Due to the nature of the data collection and accessibility via an online data platform, some entries were highly unlikely or impossible; thus, we excluded PD patients with an AAO lower than 3 years. Most of the patients were White/Caucasian (89.9%) (Table S1). PD patients had a mean age at examination (AAE) of 65.7 ± 10.2 SD years (range 13.8–119.0 years) and a mean AAO of 60.4 ± 11.0 SD years (range 5.1–115.4 years); 40.4% of PD patients were female. Patient recruitment for the Fox Insight Study has been previously described [21]. Data from a separate replication cohort of German iPD patients (EPIPARK) were used to test novel associations [22]. In the EPIPARK cohort, PD patients had a mean AAE of 67.7 ± 10.3 SD years (range 30.0–90.0 years) and a mean AAO of 54.8 ± 13.2 SD years (range 13.0–81.0 years); 37.3% of PD patients were female. Participant questionnaires are described in detail in the Supplementary text.

### Lifestyle factors

Patients were classified as tobacco users, if they smoked more than 100 cigarettes in their lifetime or if they smoked at least one cigarette per day over a minimal period of 6 months or if they used smokeless tobacco at least once per day for more than 6 months. Patients were classified as coffee consumers if they regularly drank caffeinated coffee at least once per week over a period of at least 6 months. The same classification was used for caffeinated black tea. Lastly, patients were classified as aspirin users if they took at least two pills per week over a minimum of 6 months.

Duration of smoking, caffeine consumption and aspirin intake were estimated according to the age the patients started using either substance subtracted from the age at termination. If the patients terminated the consumption after their AAO, the age the patients started was subtracted from their AAO. Periods where the patients stopped regularly consuming were not included in the duration. Smoking dosage was estimated as cigarettes smoked per day within smoking duration time excluding implausible values, so that only values lower than 100 cigarettes per day were included in the analyses. Coffee and black tea dosage was defined as cups per week the patients drank within drinking duration time, excluding all values higher than 100 cups per week from the analysis. Aspirin dosage was defined as pills per week the patients took within aspirin intake duration time. The number of cigarettes for non-smokers, cups of coffee or black tea for non-drinkers and pills per week for aspirin non-users was set to zero. The intensity of each environmental factor is estimated as dosage over duration (dosage × duration).

### Statistical analysis

For statistical analyses, non-parametric Mann–Whitney *U* test was performed to compare the distribution of AAO between different groups. For correlation analyses, non-parametric Spearman’s correlations and linear regression analyses were used to assess correlations and interactions between variables (GraphPad Software Inc., San Diego, CA, USA). Various multi-linear regression models were used to investigate the relationship between environmental factors, age, gender and potential comorbidities (IBM SPSS Statistics, Stanford, CA, USA) (details of each model are in Supplementary text). Reported *p* values remain descriptive because they are not corrected for multiple testing and results are exploratory. Patients with missing data on AAO or use of environmental and lifestyle factors were not included in the analyses.

### Regression models

#### Regression model investigating AAO, AAE, environmental factors (binary/dosage/duration)

Age is considered a risk for PD and affects the general dosage and duration of environmental factors. We applied a multiple regression model using AAO as dependent variable and AAE and each environmental factor as covariates. Environmental factors were handled in three different ways: (1) binary (yes–no indication), (2) dosage as a continuous variable, and (3) duration as a continuous variable (IBM SPSS Statistics). Including age at examination as a covariate improves the understanding of how it might influence our models (details in Supplementary text).

#### Regression model investigating AAO, AAE, gender, environmental factors (binary/dosage/duration) and comorbidities

We estimated a multiple regression model using AAO as dependent variable and further variables as covariates: AAE, gender, and each environmental factor handled in three different ways: (1) binary (yes–no indication), (2) dosage as a continuous variable, and (3) duration as a continuous variable. For the investigation of smoking and aspirin, several potential comorbidities (lung diseases; heart diseases, arthritis, back pain and surgeries with anesthesia) were explored (IBM SPSS Statistics) (details in Supplementary text).

#### Regression model investigating AAO and combined environmental factors

To evaluate whether the environmental factors show a combined effect, this multiple regression model used the AAO as dependent variable and smoking, coffee drinking and aspirin intake, all handled binary, as covariates (details in Supplementary text).

#### Regression model investigating AAO, AAE, gender, combined environmental factors and comorbidities

To evaluate potential confounders and a possible combined effect of all three environmental factors, this multiple regression model adjusted for more covariates, using the AAO as dependent variable and AAE, gender, a selected comorbidity (back pain), and smoking, coffee drinking and aspirin intake as covariates (details in Supplementary text).

### Literature review

We performed a systematic literature review for environmental factors that influence AAO in PD and searched for literature via PubMed that was published before December 14, 2021. We used the free text search terms “Parkinson onset smoking”, resulting in 221 articles, “Parkinson onset caffeine”, resulting in 46 articles, “Parkinson onset coffee”, resulting in 52 articles and “Parkinson onset aspirin”, resulting in 13 articles. Detailed descriptions of exclusion criteria are summarized in Fig. S2.

## Results

### Smoking

**Fig. 1:**
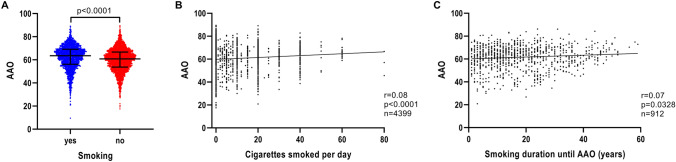
Association of AAO and tobacco use, smoking intensity and smoking duration in iPD. **a** Scatter plot of AAO of patients with iPD stratified by smoking status. Median values and interquartile ranges (IQR) are depicted. **b** Correlation between number of cigarettes smoked per day and AAO of patients with iPD. **c** Correlation between number of years of smoking until AAO and AAO of patients with iPD. *p* value: exploratory Mann–Whitney *U* test was performed for pairwise comparisons; non-parametric Spearman’s correlation and simple linear regression analyses were used to assess interactions between variables; *p* = Spearman’s exploratory *p* value, *r* = Spearman’s rank correlation coefficient

Patients with iPD, who reported use of tobacco, had a later AAO (*n* = 2148; median AAO = 63.5 years; IQR = 56.1–69.1) compared to non-users (*n* = 3375; median AAO = 60.8 years; IQR = 53.7–66.7) (*p* < 0.0001) (Fig. 1A and Table 1). Investigation of possible smoking dosage effects on AAO showed that the number of cigarettes per day was associated with later AAO (*n* = 4399, *r* = 0.08, *p* < 0.0001) (Fig. 1B), despite a small correlation strength. Similarly, a longer duration of smoking showed a positive correlation with AAO (*n* = 912, *r* = 0.07, *p* = 0.0328) (Fig. 1C), but again with a small correlation strength.

**Table 1:** Association of environmental factors and AAO

	Yes	No	*p* value
Tobacco			
*n*	2148	3375	NA
Median AAO (IQR)	63.5 (56.1–69.1)	60.8 (53.7–66.7)	< 0.0001
Coffee			
*n*	3993	1133	NA
Median AAO (IQR)	61.9 (54.7–67.6)	59.4 (52.1–65.6)	< 0.0001
Black tea			
*n*	1719	2449	NA
Median AAO (IQR)	61.0 (54.1–66.7)	61.3 (53.5–67.2)	0.8228
Aspirin			
*n*	1003	1989	NA
Median AAO (IQR)	64.0 (57.9–69.0)	59.1 (51.8–64.9)	< 0.0001

Median AAO stratified by tobacco use, coffee consumption, black tea consumption and aspirin intake

We investigated whether AAE contributed to the correlation between smoking and AAO as age is considered a risk for PD. When modeled in a linear regression to predict AAO, AAE (*p* < 1 × 10^–5^, *β* > 0.9277, SE < 0.0168), smoking (binary) (*p* = 0.0002, *β* = 0.5354, SE = 0.1424) and smoking dosage (*p* = 0.0016, *β* = 0.0172, SE = 0.0055) remained in the model as independent predictors, but the smoking duration (*p* = 0.5583, *β* = 0.0074, SE = 0.0127) did not (Table S2).

When investigating smoking, we found a correlation between dosage and duration (*p* < 1 × 10^–5^, *r* = 0.25).

To evaluate more potential predictors of AAO, we performed a sensitivity analysis. When modeled in a linear regression to predict AAO (Supplementary text), with covariates smoking binary/dosage or duration, AAE, gender, and lung diseases, smoking (binary) (*p* = 0.0005, *β* = 0.5051, SE = 0.1456) and smoking dosage (*p* = 0.0030, *β* = 0.0165, SE = 0.0055) showed a positive association with AAO. However, smoking duration (*p* = 0.5741, *β* = 0.0074, SE = 0.0131) was not found to be associated with AAO. A positive relationship for AAO with AAE (*p* < 1 × 10^–5^, *β* > 0.9254, SE < 0.0179) was also observed. We also tested for lung diseases including chronic obstructive pulmonary disease (COPD) as potential comorbidity, but these did not show any association with AAO (*p* > 0.7642, *β* > 0.0053, SE < 0.4432) (Table S2).

### Caffeine

**Fig. 2:**
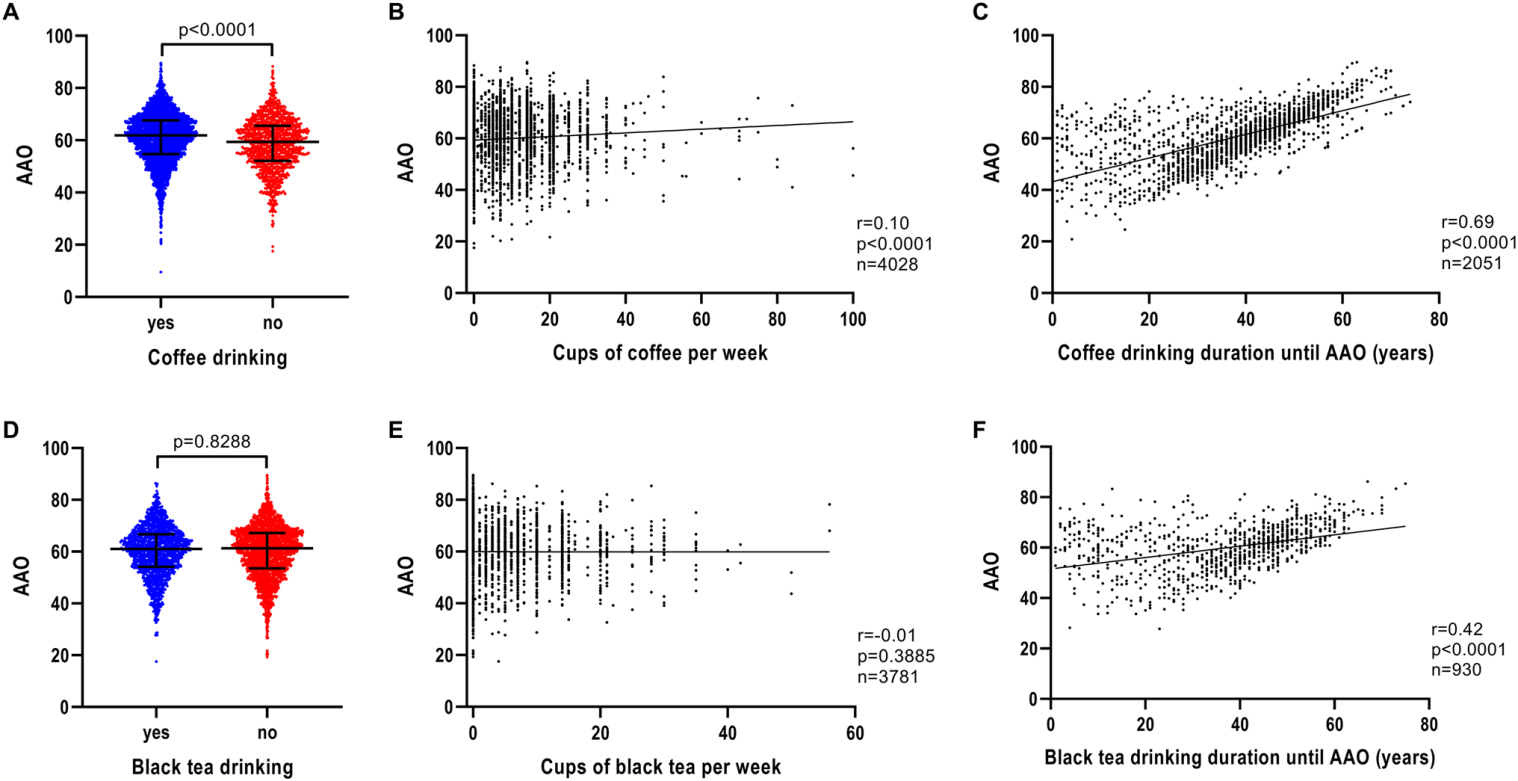
Association of AAO and caffeine consumption, caffeine drinking intensity and caffeine drinking duration in iPD. **a** Scatter plot of AAO of patients with iPD stratified by coffee consumption. Median values and interquartile ranges (IQR) are depicted. **b** Correlation between number of cups of coffee per week and AAO of patients with iPD. **c** Correlation between number of years of coffee drinking until AAO and AAO of patients with iPD. **d** Scatter plot of AAO of patients with iPD stratified by black tea consumption. **e** Correlation between number of cups of black tea per week and AAO of patients with iPD. **f** Correlation between number of years of black tea drinking until AAO and AAO of patients with iPD. *p* value: exploratory Mann–Whitney *U* test was performed for pairwise comparisons; non-parametric Spearman correlation and simple linear regression analyses were used to assess interactions between variables; *p* = Spearman’s exploratory *p* value, *r* = Spearman’s rank correlation coefficient

Patients with iPD who drank coffee regularly had a later AAO (*n* = 3993; median AAO = 61.9 years; IQR = 54.7–67.6) compared to patients with iPD who did not drink coffee at all (*n* = 1133; median AAO = 59.4 years; IQR = 52.1–65.6) (*p* < 0.0001) (Fig. 2fA and Table 1). Investigation of a possible coffee dosage effect revealed that the number of cups of coffee per week was associated with AAO, although the correlation strength was small (*n* = 4028, *r* = 0.10, *p* < 0.0001) (Fig. 2B). Longer coffee drinking duration also showed a positive correlation with AAO (*n* = 2051, *r* = 0.69, *p* < 0.0001) (Fig. 2C).

We investigated whether AAE contributed to the correlation between coffee drinking and AAO. When modeled in a linear regression to predict AAO, AAE (*p* < 1 × 10^–5^, *β* > 0.8239, SE < 0.0122), coffee drinking (binary) (*p* < 1 × 10^–5^, *β* = 0.9176, SE = 0.1704), coffee drinking dosage (*p* = 8 × 10^–5^, *β* = 0.0309, SE = 0.0078) and coffee drinking duration (*p* < 1 × 10^–5^, *β* = 0.1268, SE = 0.0083) all remained in the model as independent predictors (Table S2).

Again, coffee drinking dosage and duration were correlated (*p* < 1 × 10^–5^, *r* = 0.16).

We performed a sensitivity analysis to evaluate more potential predictors of AAO. When modeled in a linear regression to predict AAO (Supplementary text), with covariates coffee drinking binary/dosage or duration, AAE and gender, a positive relationship with coffee drinking (binary) (*p* < 1 × 10^–5^, *β* = 0.9379, SE = 0.1750), coffee drinking dosage (*p* = 0.0001, *β* = 0.0321, SE = 0.0081) and coffee drinking duration (*p* < 1 × 10^–5^, *β* = 0.1276, SE = 0.0084) was revealed. In addition, a positive relationship for AAO with AAE (*p* < 1 × 10^–5^, *β* > 0.8237, SE < 0.0125) was also observed (Table S2).

In contrast to the findings for coffee and AAO, black tea drinking was not observed to be associated with AAO (Fig. 2D and Table 1). There was also no association between the number of cups of black tea per week and AAO (*n* = 3781, *r* = − 0.01, *p* = 0.3885) (Fig. 2E). However, there was a positive correlation of black tea drinking duration with AAO (*n* = 930, *r* = 0.42, *p* < 0.0001) (Fig. 2F).

### Aspirin

**Fig. 3:**
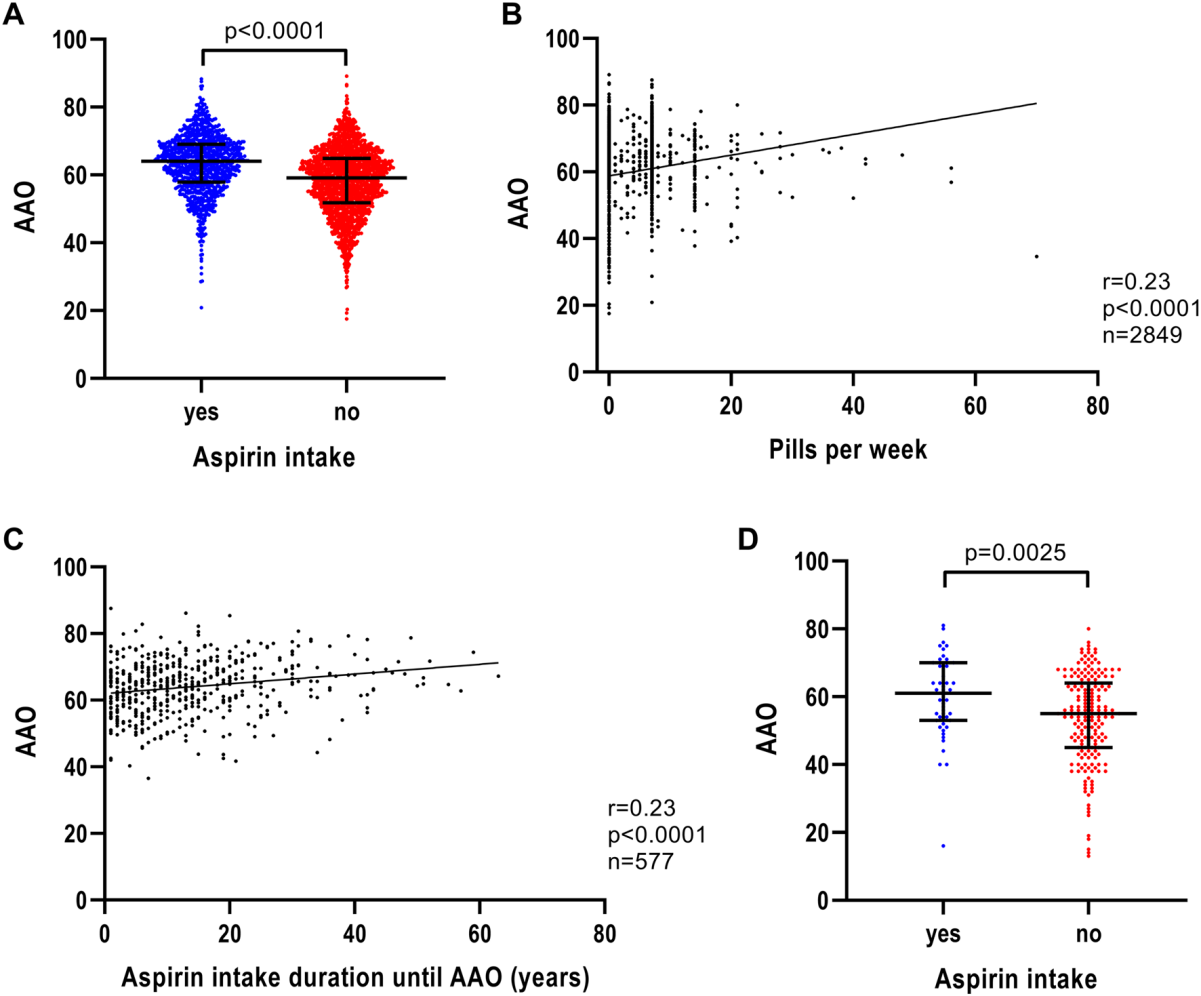
Association of AAO and aspirin intake, aspirin intake intensity and aspirin intake duration in iPD. **a** Scatter plot of AAO of patients with iPD stratified by aspirin intake. Median values and interquartile ranges (IQR) are depicted. **b** Correlation between number of aspirin pills per week and AAO of patients with iPD. **c** Correlation between number of years of aspirin intake until AAO and AAO of patients with iPD. **d** Scatter plot of AAO of patients with iPD from the EPIPARK replication cohort stratified by aspirin intake. *p* value: exploratory Mann–Whitney *U* test was performed for pairwise comparisons; non-parametric Spearman correlation and simple linear regression analyses were used to assess interactions between variables; *p* = Spearman’s exploratory *p* value, *r* = Spearman’s rank correlation coefficient

When investigating the effect of anti-inflammatory medication on AAO of patients with iPD, aspirin showed the greatest difference in AAO. Patients with iPD, who reported the use of aspirin, had a 5-year later AAO (*n* = 1003; median AAO = 64.0 years; IQR = 57.9–69.0) compared to patients who did not take aspirin (*n* = 1989; median AAO = 59.1 years; IQR = 51.8–64.9) (*p* < 0.0001) (Fig. 3A and Table 1). The difference in AAO for ibuprofen-based non-aspirin medication was small (ibuprofen users: *n* = 1087; median AAO = 60.6 years; IQR = 53.2–66.3; ibuprofen non-users: *n* = 2008; median AAO = 61.1 years; IQR = 54.2–67.0; *p* = 0.0345) or in the case of other anti-inflammatory medication we found no association at all (other anti-inflammatory drug users: *n* = 498; median AAO = 61.5 years; IQR = 54.0–66.9; other anti-inflammatory drug non-users: *n* = 2393; median AAO = 60.7 years; IQR = 53.7–66.6; *p* = 0.2495). As the association of ibuprofen was not as strong as the association with aspirin, we focused on aspirin and AAO for a more in-depth analysis.

The number of aspirin pills per week was associated with AAO (*n* = 2849, *r* = 0.23, *p* < 0.0001) (Fig. 3B). Likewise, the aspirin intake duration showed an association with AAO (*n* = 577, *r* = 0.23, *p* < 0.0001) (Fig. 3C), indicating a later AAO the longer the patients took aspirin before disease onset.

When examining the effect of AAE on aspirin intake and AAO by modeling in a linear regression to predict AAO, AAE (*p* < 1 × 10^–5^, *β* > 0.9195, SE < 0.0198), aspirin intake (binary) (*p* = 9 × 10^–5^, *β* = 0.7654, SE = 0.1958) and aspirin intake duration (*p* = 0.0165, *β* = 0.0319, SE = 0.0133) remained in the model but the aspirin dosage diminished as independent predictor (*p* = 0.0972, *β* = 0.0315, SE = 0.0190) (Table S2).

To evaluate more potential predictors of AAO, we performed a sensitivity analysis. When modeled in a linear regression to predict AAO (Supplementary text), with covariates aspirin intake binary/dosage or duration, AAE, gender, and potential comorbidities (heart diseases/arthritis/back pain/surgeries with anesthesia), aspirin intake (binary) showed a positive relationship with AAO (*p* < 0.0008, *β* > 0.6732, SE < 0.2063) as well as aspirin intake duration (*p* < 0.0153, *β* > 0.0338, SE = 0.0140). In contrast to this, aspirin intake dosage was not associated with AAO (*p* > 0.1188, *β* < 0.0303, SE < 0.0197). However, in all aspirin intake models, a positive relationship for AAO with AAE (*p* < 1 × 10^–5^, *β* > 0.9193, SE < 0.0213) was observed. In addition, in the models that included aspirin intake (binary) or aspirin intake dosage as covariate, a negative relationship for back pain with AAO (*p* < 0.0264, *β* < − 0.4144, SE < 0.1867) was found (Table S2).

### Replication cohort

Since the aspirin and PD AAO association has not been investigated and published previously, we utilized a separate German iPD cohort to investigate further. In the EPIPARK cohort, patients with iPD who reported the use of at least one aspirin pill per week over a minimal period of one month had a more than 6 year later AAO (*n* = 49; median AAO = 61.0 years; IQR = 53.0–70.0) compared to patients who did not take aspirin (*n* = 188; median AAO = 55.0 years; IQR = 45.0–64.0) (*p* = 0.0025) (Fig. 3D).

### Combined effect of smoking, coffee drinking and aspirin intake

To investigate whether there is a combined effect of smoking, coffee drinking and aspirin intake, we used a linear regression model to predict AAO (Supplementary text), showing that all three factors smoking (binary) (*p* < 1 × 10^–5^, *β* = 1.8261, SE = 0.3767), coffee drinking (binary) (*p* < 1 × 10^–5^, *β* = 2.5233, SE = 0.4158), as well as aspirin intake (binary) (*p* < 1 × 10^–5^, *β* = 4.8768, SE = 0.3698) remained in the model as an independent predictors (Table S2).

To consider more potential predictors of AAO and comorbidities, we performed a sensitivity analysis (Supplementary text). When modeled in a linear regression to predict AAO, with covariates smoking (binary), coffee drinking (binary), aspirin intake (binary), AAE, gender, and back pain, we found a positive relationship with smoking (binary) (*p* = 0.0014, *β* = 0.6400, SE = 0.2006), coffee drinking (binary) (*p* < 1 × 10^–5^, *β* = 1.1057, SE = 0.2222), aspirin intake (binary) (*p* = 0.0003, *β* = 0.7463, SE = 0.2041), AAE (*p* < 1 × 10^–5^, *β* = 0.9224, SE = 0.0109) and a negative relationship with back pain (*p* = 0.0034, *β* = − 0.5435, SE = 0.1855) (Table S2).

## Discussion

We found an association between the general intake of aspirin, number of pills per week and aspirin intake duration with later AAO. These results were additionally investigated in a multivariate linear regression model to predict AAO and revealed an association with aspirin intake when examined dichotomous as well as with aspirin intake duration, which was further validated after including more covariates and potential comorbidities. We further replicated our findings concerning aspirin in a separate German iPD cohort (EPIPARK) [22]. The effect on PD AAO was not extended to other NSAIDs in the Fox Insight cohort. The difference in AAO for ibuprofen-based non-aspirin medication was only small between users and non-users and for other anti-inflammatory medication there was no association at all with AAO. Therefore, we focused our investigations on aspirin. The clinical effect of NSAIDs is still subject to controversial discussion. While some studies indicate a protective effect of NSAIDs or at least an association with PD [19, 23], others may see a neuro-protective potential of NSAIDs but not an association with PD at the population level [24, 25]. No other studies have explored aspirin and AAO in a large iPD cohort so far (Fig. S2). In addition to the novel findings on aspirin, we replicated previous associations for smoking and caffeine with AAO in PD, summarized in a systematic literature review (Fig. S2 and Table S3) [5, 7, 8, 10–13, 15–17, 26–40]. Thus far, 25 studies analyzed the effect of tobacco or caffeine on PD AAO. These cross-sectional studies have a patient sample size ranging from *n* = 58 to 715.

This effect was further supported by multivariate regression models. When evaluating the independence of smoking and coffee drinking from AAE by pairwise correlations, it showed that smoking (binary), smoking dosage, coffee drinking (binary), coffee drinking dosage, and coffee drinking duration remained in the model as independent predictors, however, smoking duration did not. These results were robust when including more covariates in the models.

Whether smoking delays AAO is still under debate. In our literature review (Figure S2 and Table S3) ten studies showed a delay in AAO, while two studies showed an opposite effect, and nine studies did not show a directionality. Gallo et al. [5] investigated the risk for PD for smokers and non-smokers in different groups of AAO and showed that there is a prevention of PD onset. However, they argued against a delaying effect of smoking on AAO. An association with a later AAO does not necessarily indicate a causal link. Therefore, we cannot be certain whether the negative association we found in our study is caused by smoking or by other associated factors. Further studies are required to investigate the underlying cause of a later AAO in smokers. In addition, former smokers with PD and current smokers with PD need to be separated to predict a possible long-lasting effect of smoking.

Although the correlation strength for the number of cups of coffee was relatively low, the coffee drinking duration showed a strong correlation, consistent with previous studies [23, 41–43], which was also verified in the regression models. Consistent with our study, seven other studies showed that coffee drinkers have a later PD onset. However, two studies showed an opposite effect, and two studies did not report a difference in AAO (Table S3). Caffeine is the speculated reason for the protective effect of coffee. Nevertheless, black tea had a more modest association with AAO, likely due to a lower amount of caffeine, which would further explain the strong correlation between a longer black tea drinking duration and a later AAO. The effect of coffee consumption and potential long-term effects need to be investigated in further longitudinal studies.

One strength of our study was the large sample size that provided sufficient power to assess lifestyle factors and PD onset, but also allows small magnitude associations to show significance. In addition, online self-report data collections offer many possibilities to promote epidemiological research because of convenience and accessibility for the participants and researchers. A previous study compared self-reported demographic characteristics, symptoms, medical history, and PD medication use of the Fox Insight PD cohort to other in-person observational research study cohorts [44]. They found that patterns of responses to patient-reported assessments that were obtained online on the PD cohort of the Fox Insight study were similar to PD cohorts assessed in-person. Patient-reported outcomes are becoming increasingly important to research, therapeutic development and healthcare delivery, which was already investigated in another previous study on Fox Insight [45]. However, due to the self-report assessments, data may also contain more subjective perceptions that are difficult to standardize. Additionally, we were limited to the questionnaires and data collected by Fox Insight in this study. This includes the selection of environmental factors as well as the types of questions. Thus, we were unable to assess other potential protective factors and thoroughly investigated smoking, coffee and aspirin intake. In addition, we were limited to the six-month exposure determination. This period of time might not be sufficient to show a measurable delay in AAO and a higher exposure time might be needed to demonstrate a stronger effect. Nevertheless, these findings may help to acquire a better understanding of this complex disease that can be used to developed specific therapeutic strategies.

This study is a comprehensive assessment of smoking, caffeine and aspirin intake on the onset of iPD. Besides replicating previous findings in a large self-report American cohort, novel associations of aspirin use with PD AAO were observed. These findings are so far only exploratory; however, they set the stage for future longitudinal assessments on these factors and PD clinical features.

## Supplementary Information

Below is the link to the electronic supplementary material.

Supplementary file 1 (PDF 689 KB)

## Data Availability

Data used in the preparation of this article were obtained from the Fox Insight database (https://foxden.michaeljfox.org/insight/explore/fox.jsp) on 18/10/2020. For up-to-date information on the study, visit https://foxden.michaeljfox.org/insight/explore/fox.jsp.
