# Supplementary material for: Coffee, smoking and aspirin are associated with age at onset in idiopathic Parkinson’s disease
Source: J Neurol. 2022 Mar 2;269(8):4195–203. doi: 10.1007/s00415-022-11041-x (PMC9294004; doi:10.1007/s00415-022-11041-x)
Supplement: Supplementary file 1 — (PDF 689 KB) [file 415_2022_11041_MOESM1_ESM.pdf]

Supplementary Information:

Coffee, smoking and aspirin are associated with age at onset in idiopathic Parkinson's disease

Carolin Gabbert[1], MSc, Prof. Inke R. König[2], Theresa Lüth[1], MSc, Beke Kolms[1], BSc, Meike Kasten[1,3], MD, Eva-Juliane Vollstedt[1], MD, Alexander Balck[1], MD, Fox Insight Study, Anne Grünewald[1,4], PhD, Christine Klein[1], MD, Joanne Trinh[1*], PhD

[1]Institute of Neurogenetics, University of Lübeck, Lübeck, Germany
[2]Institute of Medical Biometry and Statistics, University of Lübeck, Lübeck, Germany
[3]Department of Psychiatry and Psychotherapy, University of Lübeck, Lübeck, Germany
[4]Luxembourg Centre for Systems Biomedicine, University of Luxembourg, Esch-sur-Alzette, Luxembourg

*** Correspondence:**
Joanne Trinh
University of Lübeck
Ratzeburger Allee 160
23538 Lübeck, Germany
Email: joanne.trinh@neuro.uni-luebeck.de
Tel.: +49-451-31018202

Table S1 Demographics of the Fox Insight participants

Full Cohort (n=35,963)	Patients with PD
**Male (%)**	18,349 (51.0%)
**Female (%)**	14,528 (40.4%)
**Ethnicity:**	
**White/Caucasian (%)**	32,332 (89.9%)
**Black/African American (%)**	369 (1.0%)
**American Indian/Alaska Native (%)**	393 (1.1%)
**Asian (%)**	691 (1.9%)
**Native Hawaiian/Other Pacific Islander (%)**	47 (0.1%)
**Hispanic/Latino/Spanish Origin (%)**	1,692 (4.7%)
**Mean AAO (SD)**	60.4 (11.0)
**Median AAO (IQR)**	61.3 (53.6-68.1)
**Mean AAE (SD)**	65.7 (10.2)
**Median AAE (IQR)**	66.7 (59.6-72.6)
**Mean Current Age (SD)**	66.9 (10.2)
**Median Current Age (IQR)**	68.0 (60.8-73.7)
**Mean Disease Duration until Examination (SD)**	5.3 (5.6)
**Median Disease Duration until Examination (IQR)**	3.5 (1.2-7.6)
**Mean Disease Duration until Current Age (SD)**	6.5 (5.7)
**Median Disease Duration until Current Age (IQR)**	5.0 (2.5-8.9)

Supplementary text:

Fox Insight study:

The Fox Insight study is an ongoing online, longitudinal health study of people with and without PD with targeted enrollment set to at least 125,000 individuals [1]. The data is a rich data set facilitating discovery, validation, and reproducibility in PD research. The dataset is generated through routine longitudinal assessments (health and medical questionnaires evaluated at regular cycles); one-time health and disease questionnaires about symptoms, daily activities, and other factors; and, in a subgroup of people with PD, genetic data collection. Qualified researchers can explore, analyze, and download patient-reported outcomes (PROs) data and PD-related genetic variants at https://foxden.michaeljfox.org. The full Fox Insight genetic data set, including approximately 650,000 single nucleotide polymorphisms (SNPs) per participant, can be requested separately with institutional review.

Fox Insight participants were 18 years of age or older and provided informed consent. In the process of registration, participants were divided into two groups, PD patients and controls, the latter were asked about new diagnoses every three months. PD patients responded to health, non-motor assessments, motor assessments, quality of life, and lifestyle questionnaires.

The PD-RFQ-U on "Smoking and Tobacco" questionnaire was used to evaluate smoking, the PD-RFQ-U on "Caffeine" to evaluate coffee drinking and black tea drinking, and the PD-RFQ-U on "Anti-inflammatory Medication History" for anti-inflammatory drug intake. The survey on "Your Health History" was used to include possible comorbidities in our models. All of these data were self-reported by the patients.

For each environmental or lifestyle factor the corresponding datasets were downloaded from the FoxDEN website (https://foxden.michaeljfox.org/insight/explore/fox.jsp) (log:18/10/2020).

Statistical analysis:

For a first statistical analysis, non-parametric Mann-Whitney U test was performed to compare the distribution of AAO between different groups. For correlation analyses, non-parametric Spearman correlations and linear regression analyses were used to assess correlations and interactions between variables (GraphPad Software Inc., San Diego, CA, USA). For a more in-depth analysis, we performed multilinear regression models to investigate the relationship between environmental factors, age, disease duration, motor/non-motor symptoms and potential comorbidities (IBM SPSS Statistics).

Regression model investigating AAO, AAE, environmental factors (binary/dosage/duration):

→ glm(formula = AAO ~ AAE + EnvFactorBinary, family = gaussian, data = data)
→ glm(formula = AAO ~ AAE + EnvFactorDosage, family = gaussian, data = data)
→ glm(formula = AAO ~ AAE + EnvFactorDuration, family = gaussian, data = data)

Regression model investigating AAO, AAE, gender, environmental factors (binary/dosage/duration) and comorbidities:

→ glm(formula = AAO ~ AAE + Gender + EnvFactorBinary (+Comorbidity), family = gaussian, data = data)
→ glm(formula = AAO ~ AAE + Gender + EnvFactorDosage (+Comorbidity), family = gaussian, data = data)
→ glm(formula = AAO ~ AAE + Gender + EnvFactorDuration (+Comorbidity), family = gaussian, data = data)

Regression model investigating AAO and combined environmental factors:

→ glm(formula = AAO ~ SmokingBinary + CoffeeBinary + AspirinBinary, family = gaussian, data = data)

Regression model investigating AAO, AAE, gender, combined environmental factors and comorbidities:

→ glm(formula = AAO ~ AAE + Gender + SmokingBinary + CoffeeBinary + AspirinBinary + BackPain, family = gaussian, data = data)

Exclusion and inclusion criteria:

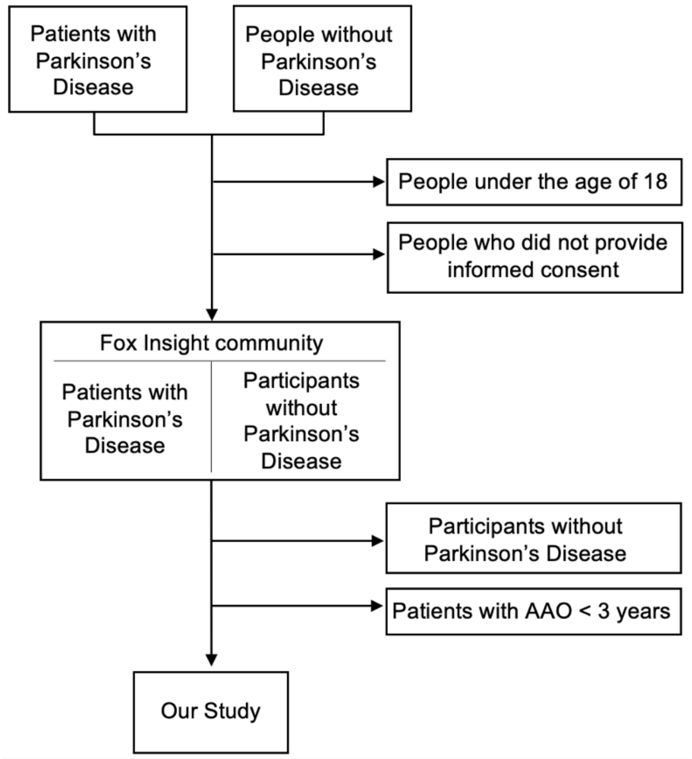

Fig. S1 Work flow of the inclusion and exclusion criteria of participant recruitment from the Fox Insight Study and for this study.

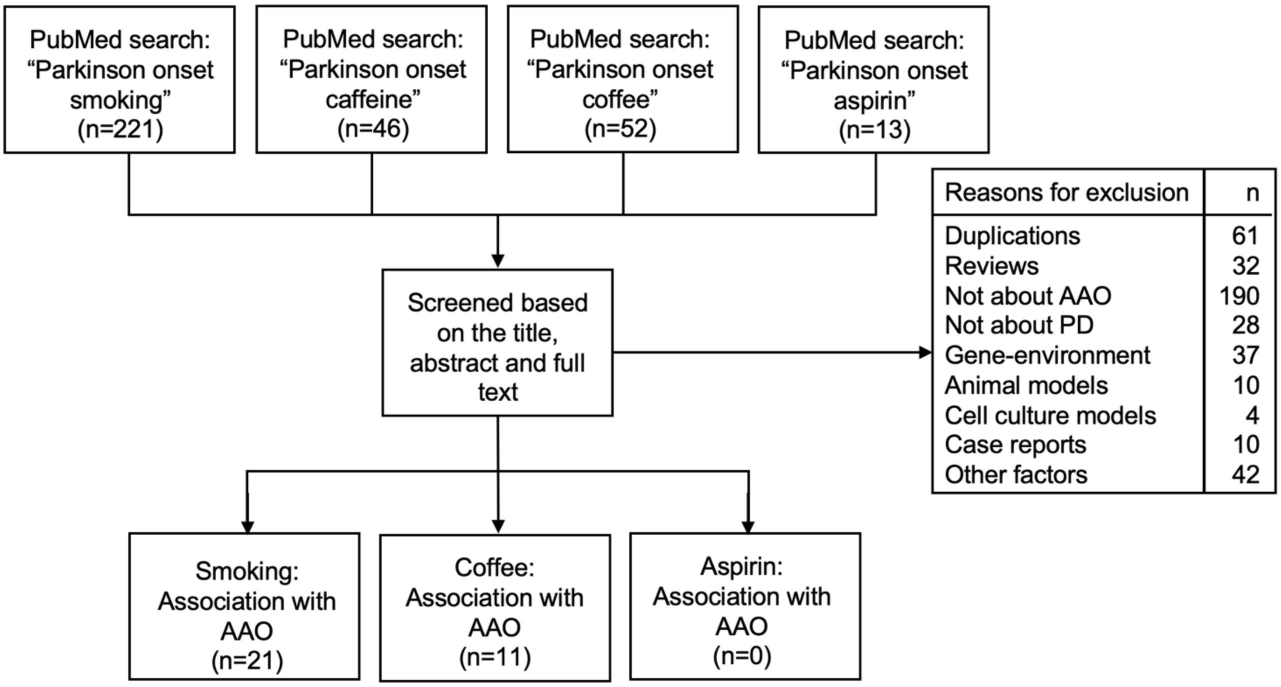

Fig. S2 Work flow of the literature search in PubMed. We searched for literature via PubMed that was published before December 14, 2021. We used the free text search terms "Parkinson onset smoking", resulting in 221 articles, "Parkinson onset caffeine", resulting in 46 articles, "Parkinson onset coffee", resulting in 52 articles and "Parkinson onset aspirin", resulting in 13 articles. These were screened based on the title, abstract and full text, excluding all articles not directly investigating smoking, coffee drinking or aspirin intake and their influence on AAO in PD. Reasons for exclusion and the number of excluded articles are shown (multiple reasons for exclusion were possible). In the end, 21 articles were obtained that described a relationship between AAO and smoking, and 11 articles describing a relationship between AAO and coffee, with an overlap of 7 articles. There was no study that investigated the association between AAO and aspirin. Reasons for exclusion: Duplications: duplicates between the different search terms; Reviews: review articles without new investigations; Not about AAO: articles not examining AAO or articles not investigating the association between AAO and smoking, caffeine/coffee or aspirin; Not about PD: articles about other diseases or about symptoms or parts of PD; Gene-environment: studies investigating gene-environment interactions and the influence of environmetal factors on specific genotypes; Animal models: articles studying animals, not humans; Cell culture models: studies only on cells; Case reports: descriptions of single cases; Other factors: studies investigating other environmental factors than smoking, coffee/caffeine or aspirin in PD or only adjusting for smoking, coffee/caffeine or aspirin in regression models to predict other factors

Table S2 Generalized linear models. Regression models for AAO and environmental factors smoking, coffee drinking and aspirin intake in the Fox Insight cohort

Dependent variable: AAO

Covariates	Regression coefficient β	Standard error	p-value
AAE	0.9343	0.0075	$<1x10^{-5}$
Smoking (binary)	0.5354	0.1424	0.0002

Covariates	Regression coefficient β	Standard error	p-value
AAE	0.9277	0.0083	$<1x10^{-5}$
Smoking Dosage	0.0172	0.0055	0.0016

Covariates	Regression coefficient β	Standard error	p-value
AAE	0.9543	0.0168	$<1x10^{-5}$
Smoking Duration	0.0074	0.0127	0.5583

Covariates	Regression coefficient β	Standard error	p-value
AAE	0.9298	0.0077	$<1x10^{-5}$
Coffee drinking (binary)	0.9176	0.1704	$<1x10^{-5}$

Covariates	Regression coefficient β	Standard error	p-value
AAE	0.9348	0.0086	$<1x10^{-5}$
Coffee drinking Dosage	0.0309	0.0078	$8x10^{-5}$

Covariates	Regression coefficient β	Standard error	p-value
AAE	0.8239	0.0122	$<1x10^{-5}$
Coffee drinking Duration	0.1268	0.0083	$<1x10^{-5}$

Covariates	Regression coefficient β	Standard error	p-value
AAE	0.9272	0.0103	$<1x10^{-5}$
Aspirin intake (binary)	0.7654	0.1958	$9x10^{-5}$

Covariates	Regression coefficient β	Standard error	p-value
AAE	0.9318	0.0103	$<1x10^{-5}$
Aspirin intake Dosage	0.0315	0.0190	0.0972

Covariates	Regression coefficient β	Standard error	p-value
AAE	0.9195	0.0198	$<1x10^{-5}$
Aspirin intake Duration	0.0319	0.0133	0.0165

Covariates	Regression coefficient β	Standard error	p-value
AAE	0.9334	0.0078	$<1x10^{-5}$
Gender	0.0234	0.1419	0.8688
Lung Disease	0.0608	0.2028	0.7642
Smoking (binary)	0.5051	0.1456	0.0005

Covariates	Regression coefficient β	Standard error	p-value
AAE	0.9254	0.0086	$<1x10^{-5}$
Gender	0.0292	0.1564	0.8518
Lung Disease	0.0332	0.2239	0.8822
Smoking Dosage	0.0165	0.0055	0.0030

Covariates	Regression coefficient β	Standard error	p-value
AAE	0.9627	0.0179	$<1x10^{-5}$
Gender	0.2611	0.3259	0.4230
Lung Disease	0.0053	0.4432	0.9905
Smoking Duration	0.0074	0.0131	0.5741

Covariates	Regression coefficient β	Standard error	p-value
AAE	0.9279	0.0080	$<1x10^{-5}$
Gender	-0.0178	0.1450	0.9026
Coffee drinking (binary)	0.9379	0.1750	$<1x10^{-5}$

Covariates	Regression coefficient β	Standard error	p-value
AAE	0.9340	0.0089	$<1x10^{-5}$
Gender	0.1385	0.1635	0.3969
Coffee drinking Dosage	0.0321	0.0081	0.0001

Covariates	Regression coefficient β	Standard error	p-value
AAE	0.8237	0.0125	$<1x10^{-5}$
Gender	0.2130	0.1900	0.2623
Coffee drinking Duration	0.1276	0.0084	$<1x10^{-5}$

Covariates	Regression coefficient β	Standard error	p-value
AAE	0.9250	0.0108	$<1x10^{-5}$
Gender	0.0306	0.1860	0.8695
Aspirin intake (binary)	0.6979	0.2063	0.0007
Heart Disease	0.0228	0.2747	0.9340

Covariates	Regression coefficient β	Standard error	p-value
AAE	0.9259	0.0109	$<1x10^{-5}$
Gender	0.0100	0.1879	0.9575
Aspirin intake (binary)	0.6791	0.2016	0.0008
Arthritis	0.0650	0.1911	0.7337

Covariates	Regression coefficient β	Standard error	p-value
AAE	0.9282	0.0107	$<1x10^{-5}$
Gender	0.0252	0.1849	0.8917
Aspirin intake (binary)	0.7082	0.2012	0.0004
Back Pain	-0.4855	0.1830	0.0080

Covariates	Regression coefficient β	Standard error	p-value
AAE	0.9264	0.0107	$<1x10^{-5}$
Gender	0.0108	0.1861	0.9538
Aspirin intake (binary)	0.6732	0.2018	0.0008
Surgeries with Anesthesia	0.1781	0.3219	0.5801

Covariates	Regression coefficient β	Standard error	p-value
AAE	0.9274	0.0109	$<1x10^{-5}$
Gender	0.0504	0.1888	0.7893
Aspirin intake Dosage	0.0263	0.0197	0.1811
Heart Disease	0.2443	0.2827	0.3875

Covariates	Regression coefficient β	Standard error	p-value
AAE	0.9292	0.0109	$<1x10^{-5}$
Gender	0.0082	0.1906	0.9657
Aspirin intake Dosage	0.0267	0.0194	0.1693
Arthritis	0.1215	0.1950	0.5331

Covariates	Regression coefficient β	Standard error	p-value
AAE	0.9319	0.0107	$<1x10^{-5}$
Gender	0.0309	0.1875	0.8693
Aspirin intake Dosage	0.0303	0.0194	0.1188
Back Pain	-0.4144	0.1867	0.0264

Covariates	Regression coefficient β	Standard error	p-value
AAE	0.9300	0.0108	$<1x10^{-5}$
Gender	0.0149	0.1887	0.9372
Aspirin intake Dosage	0.0267	0.0194	0.1703
Surgeries with Anesthesia	0.2440	0.3229	0.4499

Covariates	Regression coefficient β	Standard error	p-value
AAE	0.9193	0.0213	$<1x10^{-5}$
Gender	0.2667	0.3301	0.4191
Aspirin intake Duration	0.0353	0.0140	0.0114
Heart Disease	0.2950	0.3396	0.3850

Covariates	Regression coefficient β	Standard error	p-value
AAE	0.9204	0.0212	$<1x10^{-5}$
Gender	0.1529	0.3344	0.6474
Aspirin intake Duration	0.0338	0.0140	0.0153
Arthritis	0.3836	0.3166	0.2257

Covariates	Regression coefficient β	Standard error	p-value
AAE	0.9221	0.0210	$<1x10^{-5}$
Gender	0.2600	0.3293	0.4297
Aspirin intake Duration	0.0359	0.0140	0.0103
Back Pain	-0.3227	0.3078	0.2945

Covariates	Regression coefficient β	Standard error	p-value
AAE	0.9231	0.0210	$<1x10^{-5}$
Gender	0.2654	0.3294	0.4204
Aspirin intake Duration	0.0362	0.0140	0.0097
Surgeries with Anesthesia	-0.7760	0.7107	0.2748

Covariates	Regression coefficient β	Standard error	p-value
Smoking (binary)	1.8261	0.3767	$<1x10^{-5}$
Coffee drinking (binary)	2.5233	0.4158	$<1x10^{-5}$
Aspirin intake (binary)	4.8768	0.3698	$<1x10^{-5}$

Covariates	Regression coefficient β	Standard error	p-value
AAE	0.9224	0.0109	$<1x10^{-5}$
Gender	0.1592	0.1881	0.3975
Smoking (binary)	0.6400	0.2006	0.0014
Coffee drinking (binary)	1.1057	0.2222	$<1x10^{-5}$
Aspirin intake (binary)	0.7463	0.2041	0.0003
Back Pain	-0.5435	0.1855	0.0034

Table S3 Publications on AAO and smoking/coffee of the literature search in PubMed

Authors, Year (PMID)	n (PD/control)	n (smokers with PD/ non-smokers with PD)	n (coffee drinkers with PD/ coffee non-drinkers with PD)	Effect on AAO
Benedetti et al., 2000 (11087780) [2]	196/196	NA	NA	**Smoking:**<br>- Ever-smokers: median AAO 70 years<br>- Never-smokers: median AAO 71.5 years<br>→ Similar AAO<br><br>**Coffee:**<br>- Coffee-drinkers: median AAO 72 years<br>- Non-drinkers median AAO 64 years<br>→ Later AAO
Kandinov et al., 2009 (18434232) [3]	278/0	111/167	180/98	**Smoking:**<br>- Smokers: 1-9 pack-years: mean AAO 57.1 years, ≥10 pack-years: mean AAO 61.2 years<br>- Non-smokers: mean AAO 57.2 years ($p=0.7$; $p=0.04$)<br>- A higher amount of cigarettes smoked per day, showed a later AAO<br>→ Later AAO (when ≥10 pack-years)<br><br>**Coffee:**<br>- Coffee-drinkers: <2 daily cups: mean AAO 58 years, 2-3 daily cups: mean AAO 57.6 years, >3 daily cups: mean AAO 55 years<br>- Coffee non-drinkers: mean AAO 59.5 years<br>→ Earlier AAO (with dosage effect)

Lüth et al., 2020 (32875616) [4]	342/57<br><br>(142 with LRRK2 G2019S PD) and 57 mutation carriers)	112 /199<br><br>(41 smokers with LRRK2 G2019S PD and 85 non-smokers with LRRK2 G2019S PD)	182 /130<br><br>(62 coffee drinkers with LRRK2 G2019S PD and 63 coffee non-drinkers with LRRK2 G2019S PD)	**Smoking:**<br>- LRRK2 PD:<br>Smokers: median AAO 60 years<br>Non-smokers: median AAO 52 years (p=0.0215)<br>- iPD:<br>Smokers: median AAO 55 years,<br>Non-smokers: median 53.5 years (p=0.7906)<br>- Number of cigarettes per day correlated with AAO (p=0.0296) as well as smoking duration (p<0.0001)<br>→ Later AAO (in LRRK2 PD)<br><br>**Coffee:**<br>- LRRK2 PD:<br>Coffee-drinkers: median AAO 55 years<br>Non-drinkers: median AAO 52 years (p=0.5439)<br>- iPD:<br>Coffee-drinkers: median AAO 55 years<br>Non-drinkers: median AAO 52 years (p=0.3279)<br>→ Trend to a later AAO, but no significant difference
Maher et al., 2002 (11781409) [5]	396/0	81/337	401/17	**Smoking:**<br>- Among siblings who smoked, pack-years of smoking was related to later age at onset (p=0.0001)<br>→ Later AAO<br><br>**Coffee:**<br>- The mean age at onset did not differ according to exposure to coffee (p=0.79)<br>→ Similar AAO

Wijeyekoon et al., 2017 (29057010) [6]	144/102	NA	NA	**Smoking:**<br>- Ever-smoking in males associated with delayed AAO (p=0.048)<br>→ Later AAO (in males)<br><br>**Coffee:**<br>- Regular coffee drinking associated with later AAO (p<0.001)<br>→ Later AAO
Wilk et al., 2007 (17408493) [7]	NA	NA	NA	**Smoking:**<br>- Current cigarette smoking is a predictor of PD age-at-onset and age at-enrollment in both the full and age-restricted samples.<br>→ Later AAO<br><br>**Coffee:**<br>- Coffee drinking is a predictor of PD age-at-onset and age at-enrollment in both the full and age-restricted samples.<br>→ Later AAO
Yahalom et al., 2020 (32310186) [8]	225/0<br><br>(65 with LRRK2 G2019S PD and 60 with GBA N370S PD)	98 /127<br><br>(26 with LRRK2 G2019S PD and 39 mutation carriers and 27 with GBA N370S PD and 33 mutation carriers)	199 /25<br><br>(56 with LRRK2 G2019S PD and 9 mutation carriers and 53 with GBA N370S PD and 6 mutation carriers)	**Smoking:**<br>- Smoking associated with AAO (p = 0.032)<br>→ Later AAO<br><br>**Coffee:**<br>- Consumption level of coffee (p=0.001) significantly associated with PD AAO<br>→ Later AAO the higher the amount of coffee
De Reuck et al., 2005 (15792818) [9]	512/0	184/328	NA	**Smoking:**<br>- Ever-smokers: mean AAO 65.9 years<br>- Never-smokers: mean AAO 62.4 years (p=0.001)<br>→ Later AAO

Gallo et al., 2019 (30462234) [10]	715/213,818	312/404	NA	**Smoking:**<br>- The risk does not vary over the follow-up period, and this argues against a delaying effect of smoking on PD onset
Gigante et al., 2017 (28988683) [11]	262/0	111/151	NA	**Smoking:**<br>- Ever-smokers: mean AAO 61.7 years<br>- Never-smokers: mean AAO 59.3 years (p=0.03)<br>→ Later AAO
Grandinetti et al., 1994 (8209872) [12]	58/0	25/33	NA	**Smoking:**<br>- Ever-smokers: mean AAO 69.2 years<br>- Never-smokers: mean AAO 70.2 years (p=0.49)<br>→ Similar AAO
Greenbaum et al., 2013 (22884254) [13]	677/0	239/438	NA	**Smoking:**<br>- Ever-smokers: mean AAO 54.4 years<br>- Never-smokers: mean AAO 55.8 years (p=NS)<br>→ Similar AAO
Haack et al., 1981 (7304554) [14]	237/474	87/150	223/14<br>(coffee or tea)	**Smoking:**<br>- Smokers: mean AAO 52.7 years (men: 54.4 years, women: 42.5)<br>- Non-smokers: mean AAO 57.8 years (men: 58.9 years, women: 55.4)<br>→ Earlier AAO
Jiménez-Jiménez et al., 1992 (1484528) [15]	128/256	NA	NA	**Smoking:**<br>- No significant correlation between premorbid levels of exposure to cigarette smoking and AAO in the PD group.
Kuopio et al., 1999 (10584666) [16]	123/246	NA	NA	**Smoking:**<br>- Ever-smokers: AAO 65.0 years<br>- Never-smokers: AAO 61.8 years (p=0.051)<br>→ Later AAO<br><br>- Male ever-smokers: AAO 64.8 years<br>- Male never-smokers: AAO 58.3 years (p=0.009)<br>→ Later AAO

Martínez-Rumayor et al., 2009 (19695769) [17]	247/0	91/156	NA	**Smoking:**<br>- Smokers: mean AAO 55 years<br>- Non-smokers: mean AAO 59 years (p=0.08)<br>→ Later AAO
Mayeux et al., 1994 (8196685) [18]	150/180	69/81	NA	**Smoking:**<br>- Smokers: AAO 64.2 years<br>- Non-smokers: AAO 64.5 years (p=0.88)<br>→ Similar AAO
Neshige et al., 2021 (34130061) [19]	110/110	67/43	NA	**Smoking:**<br>- Ever-smokers: AAO 63 years<br>- Non-smokers: AAO 65 years (p=0.28)<br>→ Similar AAO
Papapetropoulos et al., 2005 (15747366) [20]	113/0	58/55 (14 smokers and 44 ex-smokers)	NA	**Smoking:**<br>- Smokers: AAO 58.6 years<br>- Ex-smokers: AAO 64.9 years<br>- Non-smokers: AAO 65.5 years (p=0.006 and p=0.011)<br>→ Earlier AAO
Scott et al., 2005 (15699372) [21]	143/168	NA	NA	**Smoking:**<br>- Ever-smokers: mean AAO 55.7 years<br>- Non-smokers: mean AAO 55.4 years<br>- 140 sibships<br>→ Similar AAO
Weisskopf et al., 2007 (17266085) [22]	137/466	128/153 (16 current smokers and 112 past smokers)	NA	**Smoking:**<br>- Current smokers: AAO 65.3 years<br>- Past smokers: AAO 67.1 years<br>- Never-smokers: AAO 66.7 years<br>→ Similar AAO

Cho et al., 2018 (29449185) [23]	196/0	NA	136/60	**Coffee:**<br>- Coffee drinkers: mean AAO 63.4 years<br>- Non-coffee drinkers: mean AAO 67.3 years (p=0.008)<br>→ Earlier AAO
Cho et al. 2019 (31412802) [24]	284/0	NA	204/80<br>Males: 120/27<br>Females: 84/53	**Coffee:**<br>- Coffee drinkers: AAO 62.8 years<br>- Non-coffee drinkers: AAO 67.0 years (p=0.001)<br>→ Earlier AAO<br><br>- Male coffee drinkers: AAO 62.6 years<br>- Male non-coffee drinkers: AAO 69.8 years (p=0.001)<br>→ Earlier AAO<br><br>- Female coffee drinkers: AAO 63.2 years<br>- Female non-coffee drinkers: AAO 65.5 years (p=NS)<br>→ Similar AAO
Gigante et al., 2018 (29362953) [25]	83/0	12/71	71/12	**Coffee:**<br>- Number of coffee drinking years associated with a significant increase in AAO (p<0.001)<br>→ Later AAO (dosage effect)
Tan et al. 2007 (18075470) [26]	418/468	81/337	401/17	**Coffee:**<br>- Significant association between caffeine intake and the onset of PD ($p=2.01 \times 10^{-5}$)<br>- Dosage effect, showing a later AAO the higher the caffeine consumption<br>→ Later AAO (with dosage effect)

References

1. Smolensky L, Amondikar N, Crawford K, Neu S, Kopil CM, Daeschler M, Riley L, andMe Research T, Brown E, Toga AW, Tanner C (2020) Fox Insight collects online, longitudinal patient-reported outcomes and genetic data on Parkinson's disease. Sci Data 7:67 https://doi.org/10.1038/s41597-020-0401-2

2. Benedetti MD, Bower JH, Maraganore DM, McDonnell SK, Peterson BJ, Ahlskog JE, Schaid DJ, Rocca WA (2000) Smoking, alcohol, and coffee consumption preceding Parkinson's disease: a case-control study. Neurology 55:1350-1358

3. Kandinov B, Giladi N, Korczyn AD (2009) Smoking and tea consumption delay onset of Parkinson's disease. Parkinsonism Relat Disord 15:41-46

4. Luth T, Konig IR, Grunewald A, Kasten M, Klein C, Hentati F, Farrer M, Trinh J (2020) Age at Onset of LRRK2 p.Gly2019Ser Is Related to Environmental and Lifestyle Factors. Mov Disord 35:1854-1858

5. Maher NE, Golbe LI, Lazzarini AM, Mark MH, Currie LJ, Wooten GF, Saint-Hilaire M, Wilk JB, Volcjak J, Maher JE, Feldman RG, Guttman M, Lew M, Waters CH, Schuman S, Suchowersky O, Lafontaine AL, Labelle N, Vieregge P, Pramstaller PP, Klein C, Hubble J, Reider C, Growdon J, Watts R, Montgomery E, Baker K, Singer C, Stacy M, Myers RH (2002) Epidemiologic study of 203 sibling pairs with Parkinson's disease: the GenePD study. Neurology 58:79-84

6. Wijeyekoon R, Suriyakumara V, Gamage R, Fernando T, Jayasuriya A, Amarasinghe D, Gunasekara H, Sirisena D, Amaratunga D, Muthukuda C, Barker RA, Williams-Gray C, De Silva R (2017) Associations between Lifestyle Factors and Parkinson's Disease in an Urban Sri Lankan Clinic Study. Int Arch Med 10

7. Wilk JB, Lash TL (2007) Risk factor studies of age-at-onset in a sample ascertained for Parkinson disease affected sibling pairs: a cautionary tale. Emerg Themes Epidemiol 4:1

8. Yahalom G, Rigbi A, Israeli-Korn S, Krohn L, Rudakou U, Ruskey JA, Benshimol L, Tsafnat T, Gan-Or Z, Hassin-Baer S, Greenbaum L (2020) Age at Onset of Parkinson's Disease Among Ashkenazi Jewish Patients: Contribution of Environmental Factors, LRRK2 p.G2019S and GBA p.N370S Mutations. J Parkinsons Dis 10:1123-1132

9. De Reuck J, De Weweire M, Van Maele G, Santens P (2005) Comparison of age of onset and development of motor complications between smokers and non-smokers in Parkinson's disease. J Neurol Sci 231:35-39

10. Gallo V, Vineis P, Cancellieri M, Chiodini P, Barker RA, Brayne C, Pearce N, Vermeulen R, Panico S, Bueno-de-Mesquita B, Vanacore N, Forsgren L, Ramat S, Ardanaz E, Arriola L, Peterson J, Hansson O, Gavrila D, Sacerdote C, Sieri S, Kuhn T, Katzke VA, van der Schouw YT, Kyrozis A, Masala G, Mattiello A, Perneczky R, Middleton L, Saracci R, Riboli E (2019) Exploring causality of the association between smoking and Parkinson's disease. Int J Epidemiol 48:912-925

11. Gigante AF, Martino T, Iliceto G, Defazio G (2017) Smoking and age-at-onset of both motor and non-motor symptoms in Parkinson's disease. Parkinsonism Relat Disord 45:94-96

12. Grandinetti A, Morens DM, Reed D, MacEachern D (1994) Prospective study of cigarette smoking and the risk of developing idiopathic Parkinson's disease. Am J Epidemiol 139:1129-1138

13. Greenbaum L, Rigbi A, Lipshtat N, Cilia R, Tesei S, Asselta R, Djaldetti R, Goldwurm S, Lerer B (2013) Association of nicotine dependence susceptibility gene, CHRNA5, with Parkinson's disease age at onset: gene and smoking status interaction. Parkinsonism Relat Disord 19:72-76

14. Haack DG, Baumann RJ, McKean HE, Jameson HD, Turbek JA (1981) Nicotine exposure and Parkinson disease. Am J Epidemiol 114:191-200
15. Jimenez-Jimenez FJ, Mateo D, Gimenez-Roldan S (1992) Premorbid smoking, alcohol consumption, and coffee drinking habits in Parkinson's disease: a case-control study. Mov Disord 7:339-344
16. Kuopio AM, Marttila RJ, Helenius H, Rinne UK (1999) Environmental risk factors in Parkinson's disease. Mov Disord 14:928-939
17. Martinez-Rumayor A, Arrieta O, Sotelo J, Garcia E (2009) Female gender but not cigarette smoking delays the onset of Parkinson's disease. Clin Neurol Neurosurg 111:738-741
18. Mayeux R, Tang MX, Marder K, Cote LJ, Stern Y (1994) Smoking and Parkinson's disease. Mov Disord 9:207-212
19. Neshige S, Ohshita T, Neshige R, Maruyama H (2021) Influence of current and previous smoking on current phenotype in Parkinson's disease. J Neurol Sci 427:117534
20. Papapetropoulos S, Singer C, Villar JM, Gonzalez J, Mash DC (2005) Does cigarette smoking provide clinically significant neuroprotection among patients diagnosed with Parkinson's disease? Mov Disord 20:641-642
21. Scott WK, Zhang F, Stajich JM, Scott BL, Stacy MA, Vance JM (2005) Family-based case-control study of cigarette smoking and Parkinson disease. Neurology 64:442-447
22. Weisskopf MG, Grodstein F, Ascherio A (2007) Smoking and cognitive function in Parkinson's disease. Mov Disord 22:660-665
23. Cho BH, Choi SM, Kim JT, Kim BC (2018) Association of coffee consumption and non-motor symptoms in drug-naive, early-stage Parkinson's disease. Parkinsonism Relat Disord 50:42-47
24. Cho BH, Choi SM, Kim BC (2019) Gender-dependent effect of coffee consumption on tremor severity in de novo Parkinson's disease. BMC Neurol 19:194
25. Gigante AF, Asabella AN, Iliceto G, Martino T, Ferrari C, Defazio G, Rubini G (2018) Chronic coffee consumption and striatal DAT-SPECT findings in Parkinson's disease. Neurol Sci 39:551-555
26. Tan EK, Chua E, Fook-Chong SM, Teo YY, Yuen Y, Tan L, Zhao Y (2007) Association between caffeine intake and risk of Parkinson's disease among fast and slow metabolizers. Pharmacogenet Genomics 17:1001-1005
